# Thermostability study of virulent Newcastle disease viruses isolated in Southern Angola

**DOI:** 10.4102/ojvr.v91i1.2147

**Published:** 2024-04-15

**Authors:** António Neto, Ana M. Henriques, Teresa Fagulha, Miguel Fevereiro

**Affiliations:** 1 Laboratory of Virology, National Institute of Agricultural and Veterinary Research, Oeiras, Portugal; 2 Laboratory for the Production of Veterinary Vaccines, Veterinary Research Institute, Lubango, Angola

**Keywords:** Angola, haemagglutinin activity, infectivity, Newcastle disease virus, thermostability

## Abstract

Newcastle disease (ND) is endemic in Angola. Several outbreaks of ND occurred in small backyard flocks and village chickens with high mortality in the southern provinces of the country, Cunene, Namibe and Huíla, in 2016 and 2018. In those years, 15 virulent ND virus (NDV) strains were isolated and grouped within subgenotype 2 of genotype VII (subgenotype VII.2). We now present a study on the thermostability of the isolates, aiming at the selection of the most thermostable strains that, after being genetically modified to reduce their virulence, can be adapted to the production of vaccines less dependent on cold chain and more adequate to protect native chickens against ND. Heat-inactivation kinetics of haemagglutinin (Ha) activity and infectivity (I) of the isolates were determined by incubating aliquots of virus at 56 °C for different time intervals. The two isolates from Namibe province showed a decrease in infectivity of 2 log_10_ in ≤ 10 min, therefore belonging to the I-phenotype, but while the NB1 isolate from 2016 maintained the Ha activity up to 30 min and was classified as thermostable virus (I^−^Ha^+^), the Ha activity of the 2018 NB2 isolate decreased by 2 log_2_ in < 30 min, being classified as a thermolabile virus (I^−^Ha^−^). Of the 13 NDV isolates from Huíla province, 10 isolates were classified as thermostable, eight with phenotype I^+^Ha^+^ and 2 with phenotype I^−^Ha^+^. The other three isolates from this province were classified as thermolabile viruses (I^−^Ha^−^).

**Contribution:**

This study will contribute to the control and/or eradication of Newcastle disease virus in Angola. The thermostable viral strains isolated from chickens in the country can be genetically manipulated by reverse genetic technology in order to reduce their virulence and use them as a vaccine in the remote areas of Angola.

## Introduction

Newcastle disease (ND) is a highly contagious, often fatal viral disease of birds. In Africa, ND is one of the major transboundary animal diseases with great impact in poultry production especially small flocks of village chickens and one of the major constraints in poverty alleviation in many low-income countries (Alders 2014). Newcastle disease virus, also known as avian *Paramyxovirus 1,* is now designated avian *orthoavulavirus-1*, one of the eight virus species belonging to the genus *Orthoavulavirus* in the *Paramyxoviridae* family (Dimitrov et al. 2019). Newcastle disease virus are single-stranded, negative-sense ribonucleic acid (RNA) viruses. Clinical presentation of ND can be difficult to distinguish from disease caused by avian influenza virus (AIV). Only through laboratory tests can a correct differential diagnosis be made between these two diseases, as well as to detect mixed infections (Miller & Torchetti 2014).

Based on the severity of the clinical signs and lesions, NDV strains are usually grouped into three pathotypes designated lentogenic (low or avirulent), mesogenic (intermediately virulent) and velogenic (highly virulent). The latter may cause near 100% mortality in non-vaccinated poultry (Alexander & Allan 1974).

Besides classification into pathotypes, NDV strains can also be classified into one of three phenotypes, according to thermostability of infectivity (I) and haemagglutinin (Ha). The three phenotypes are I^−^Ha^−^, infectivity and haemagglutinin thermolabile; I^+^Ha^+^, infectivity and haemagglutinin thermostable and I^−^Ha^+^, infectivity thermolabile and haemagglutinin thermostable (Lomniczi 1975).

Newcastle disease was first diagnosed in Angola in 1957 (Sousa 1973). In recent years, several outbreaks of the disease have occurred in free-range and backyard flocks in the southern provinces of Huíla, Namibe and Cunene, aggravating the precarious food situation of the populations of these provinces, already experiencing successive years of severe drought.

Vaccination is a common practice for the prevention and control of the disease in the poultry industry. Unfortunately, commercial ND vaccines require continuous storage at 2 °C – 8 °C and are produced in large doses per vial, making them unsuitable for small free-range flocks of chickens reared in rural areas. To avoid these disadvantages, in many countries of Africa and Asia, the avirulent thermostable Australian strains I2 and V4 have been used in the production of vaccines locally to protect village chickens against ND (Spradbrow 1993). The thermostable I-2 strain has been used to produce the NDV vaccine in Angola but in a small number of batches because of the difficulty in obtaining fertile eggs in quantity and quality. The country is now looking to obtain local NDV strains, which can serve as new virus master seed to include into the production chain of better tailored vaccines to protect native chickens.

In this article, we report the screening of 86 chicken samples for the presence of NDV and the isolation of the 15 positive isolates obtained, which were grouped within subgenotype 2 of genotype VII (subgenotype VII.2) in another study (Henriques et al. 2023). We also report the thermostability study of those isolates, in order to identify thermostable Newcastle disease virus strains able to be used as vaccine strains in the remote areas of Angola.

## Research methods and design

### Sample collection

**FIGURE 1:**
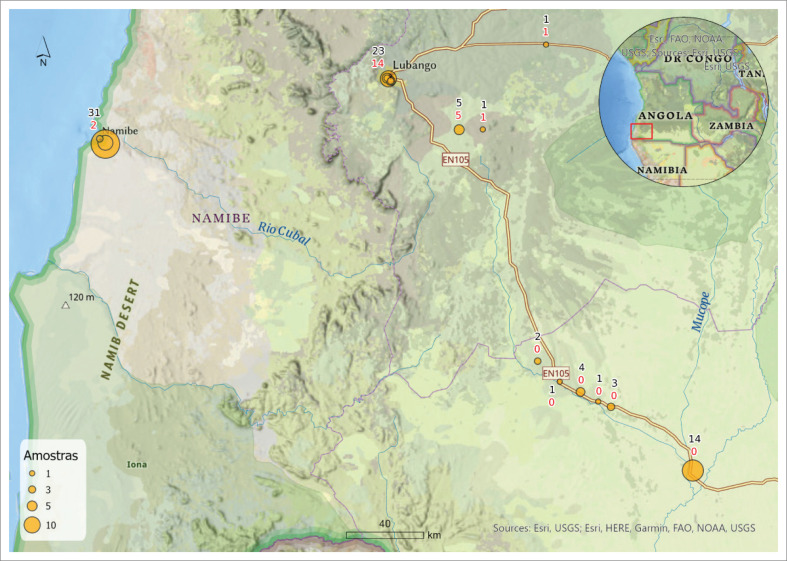
Location where samples were collected from chickens in Huíla, Namibe and Cunene provinces for virus screening. The number of samples are indicated in black, while numbers in red correspond to NDV positive samples.

The sampling focused on three southern provinces of Angola where high mortality had occurred in village chickens in the last years. Oropharyngeal and cloacal swabs as well as organs were collected from sick or recently dead chickens, found in small villages and live bird markets on the outskirts of the cities of Lubango – Huíla (*n* = 30), Namibe – Namibe (*n* = 31) and Ondjiva – Cunene (*n* = 25) (Figure 1). Except for one sample from Namibe, collected in 2016, all other samples were collected in 2018. Cotton-tipped plastic swabs and tissue samples were placed in tubes or leak proof vials containing phosphate buffered saline (PBS) with antibiotics (200 U/mL penicillin, 200 µg/mL streptomycin and 100 µg/mL gentamycin) in a proportion of 10%. Samples were transported to the laboratory as soon as it was possible in cool boxes. To reduce the risk of cross-contamination during sample collection, disposable individual protective equipment was changed between each of the locals of sampling.

### Viral RNA detection

Total RNA was extracted using a nucleic acid extraction workstation BioSprint 96 (Qiagen, Hilden, Germany), according to the manufacturer’s instructions. All samples were initially screened for NDV by L gene real-time reverse-transcription polymerase chain reaction (PCR) (Lgene/RT-qPCR) (Fuller et al. 2010). The reaction was performed in a final volume of 25 µL with 500 ng of total RNA, 25 pmol of each primer and 5 pmol of probe, using AgPath-ID One-Step RT-PCR kit (ThermoFisher Scientific, Waltham, United States [US]), according to the manufacturer’s protocol. The amplification programme for Lgene/RT-qPCR included reverse transcription for 15 min at 45 °C and activation of Taq polymerase at 95 °C for 10 min, followed by 45 cycles of denaturation at 95 °C for 10 s, annealing at 50 °C for 30 s and extension at 72 °C for 30 s. The NDV RT-qPCR positive samples were subjected to virus isolation.

The samples were also screened for AIV, using the AIV-matrix/RT-qPCR method described by Spackman and colleagues (Spackman et al. 2002).

### Virus propagation

For virus isolation, supernatants from swabs and homogenised organs were clarified by centrifugation at 1500 × g for 10 min at 5 °C. The supernatants were collected and pooled per individual bird and treated with antibiotics for 2 h at room temperature prior inoculation (0.2 mL) into the chorioallantoic cavity of five 10-day-old embryonating specific pathogen free (SPF) eggs (Lohman Tierzucht, GmbH, Cuxhaven, Germany). The inoculated eggs were incubated at 37 °C for 5 days and candled daily. When embryo deaths were first observed, by the absence of blood vessels and embryo movement, the eggs were cooled. The allantoic fluid was harvested and kept at 4 °C until tested for haemagglutination (Ha) activity and infectivity. All experiments were conducted in biosafety level 3 (BSL-3) facilities (National Reference Laboratory – Animal Health, National Institute for Agricultural and Veterinary Research [INIAV], Oeiras, Portugal).

### Newcastle disease virus thermostability and infectivity assays

Heat-inactivation kinetics of Ha activity and infectivity of the NDV isolates were determined at 56 °C. For all virus isolates, ten 0.5 mL aliquots of undiluted allantoic fluid were sealed in air-tight vials. One vial was left on ice while the other nine were incubated with agitation in a water bath kept at 56 °C (Wen et al. 2016). At regular intervals of 5 min, 10 min, 15 min, 20 min, 30 min, 40 min, 45 min, 50 min and 60 min, the vials were removed and chilled quickly on ice-cold water bath to stop heat inactivation. The Ha titer for all aliquots was determined by standard Ha assay in 96-well microplates (World Organisation for Animal Health [WOAH] 2021). In addition, heat-treated virus for 0 min, 5 min, 10 min, 15 min and 30 min was assayed by endpoint infectivity assay (TCID50). The TCID50 values were calculated using the Spearman and Kärber method (Kärber 1931; Spearman 1908). Of a serial dilution from 10^−1^ µL to 10^−9^ µL, 50 µL of each dilution were added in duplicate to BHK-21 cells in the absence of trypsin. The 96-well microplates were incubated at 37 °C in a humidified atmosphere with 5% CO_2_ and then observed microscopically for cytopathic effects after 5 days.

The thermostability of NDV isolates is shown as the mean time required for 2 log_2_ and 2 log_10_ decreases in Ha activity and infectivity, respectively. The exponential decline in the activity of Ha and infectivity are also presented as rate constants (k) with thermostable isolates showing the lowest k constants (Lomniczi 1975). The experiments described here were repeated two times. The lentogenic strain Ulster 2C of NDV, known as thermostable, was included in the assays described here, as a positive control.

A fragment of the HN protein, indicated as a major determinant of thermostability (Wen et al. 2016) was amplified using primers HN-F (5’- CGTACAGCAAGCCATCCTATC - 3’) and HN-R (5’ - GTGAAGGCATTAAATGTATAGGGA - 3’) designed for this study. The reaction was performed in a final volume of 25 µL with 500 ng of total RNA and 25 pmol of each primer, using AgPath-ID One-Step RT-PCR kit (ThermoFisher Scientific, Waltham, US), according to the manufacturer’s protocol. The amplification programme included reverse-transcription for 15 min at 45 °C and activation of Taq polymerase at 95 °C for 10 min, followed by 50 cycles of denaturation at 95 °C for 30 s, annealing at 60 °C for 30 s and extension at 72 °C for 30 s. The obtained fragment of 240 base pairs (bp) was separated by agarose gel electrophoresis, excised and purified using NZYGelpure kit (NZYTech, Lisboa, Portugal). Deoxyribonucleic acid (DNA) sequencing was performed using a BigDye Terminator cycle sequencing kit (Applied Biosystems, Foster City, CA, US) according to the manufacturer’s instructions, with primers HN-F and HN-R used in the amplification reaction. The nucleotide sequences of the amplicons were determined on an automated 3130 Genetic Analyzer system (Applied Biosystems) and assembled with SeqScape version 2.5 software (Applied Biosystems). The nucleotide sequences obtained are available in GenBank with accession numbers MN918460 to MN918474.

### Ethical considerations

Ethical clearance to conduct this study was obtained from the University of Lisbon Faculty Veterinary Medicine Ethics Committee.

## Results and discussion

### Virus detection and isolation

Clinical signs observed in the chickens in this study were watery green diarrhoea, lethargy, paralysis and torticollis. The macroscopic lesions consisted mainly of haemorrhages in the gastrointestinal tract and trachea. Collected samples were initially screened for NDV and AIV by RT-qPCR and positive samples were inoculated in SPF eggs for virus isolation. Of the 30 samples received from Huíla, 21 (70.0%) were positive by Lgene/RT-qPCR, and of these, 13 (61.9%) were positive for virus isolation. From the 31 samples from Namibe province, two tested Lgene/RT-qPCR positive (6.5%), one collected in 2016 and the other in 2018. Newcastle disease virus was successfully isolated from both samples. All samples from Cunene province (*n* = 25) were negative by Lgene/RT-qPCR and virus isolation. The absence of ND clinical cases and deaths in chickens in Cunene province is surprising, but previous outbreaks of the disease and the severe drought that ravaged the province may have reduced the population density of chickens, thus contributing of diminishing the spread of NDV.

The screening for avian influenza by AIV-matrix/RT-qPCR resulted negative in all samples from the three provinces.

The 2016 NDV isolate from Namibe province is genetically related to an outbreak that affected the neighbouring country Namibia in that year, while the nucleotide sequence of the 2018 isolate is linked to the outbreak of the same year in the province of Huíla (Henriques et al. 2023; Molini et al. 2017). The cleavage sites of the 15 isolates determined in another study (Henriques et al. 2023), showed characteristic cleavage site motif (^112^RRQKRF^117^) for the highly pathogenic strains of NDV (Bulbule et al. 2015).

### Heat-inactivation kinetics

**FIGURE 2:**
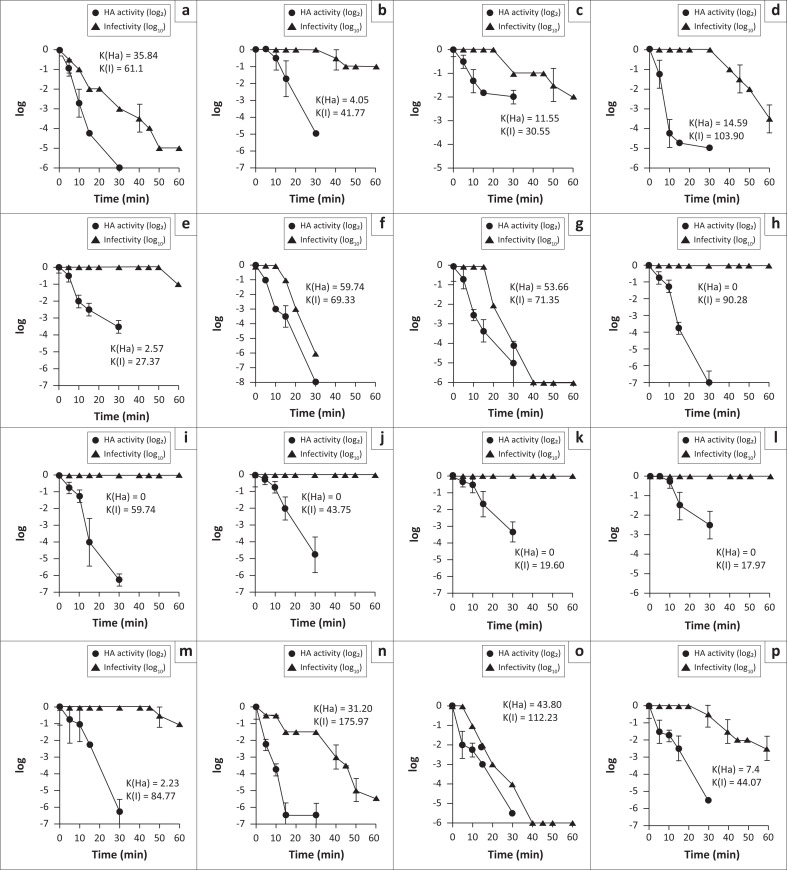
Heat-inactivation kinetics of Ha activity (▲) and infectivity (●) of the NDV isolates determined at 56 °C. The thermostability of NDV isolates is shown as the mean time required for 2 log_2_ and 2 log_10_ decreases in Ha activity and infectivity, respectively. The rate constants K(Ha) and K(I) are indicated as a measure of the exponential decline in activity of Ha and infectivity, respectively. Data shown represented the average of two independent experiments (Mean ± s.d., *n* = 2). The lentogenic strain Ulster 2C of NDV, known as thermostable, was also included. (a) AAvV-1/chicken/AO-HL1/2018; (b) AAVV-1/chicken/AO-HL2/2018; (c) AAVV-1/chicken/AO-HL3/2018; (d) AAVV-1/chicken/AO-HL4/2018; (e) AAvV-1/chicken/AO-HL5/2018; (f) AAvV-1/chicken/AO-HL6/2018; (g) AAvV-1/chicken/AO-HL7/2018; (h) AAvV-1/chicken/AO-HL8/2018; (i) AAvV-1/chicken/AO-HL9/2018; (j) AAvV-1/chicken/AO-HL10/2018; (k) AAVV-1/chicken/AO-HL11/2018; (l) AAVV-1/chicken/AO-HL12/2018; (m) AAVV-1/chicken/AO-HL13/2018; (n) AAVV-1/chicken/AO-NB1/2016; (o) AAVV-1/chicken/AO-NB2/2018 and (p) ULSTER 2C.

The mean time for 2 log decrease in infectivity and Ha activity of the NDV field isolates was determined and is indicated in (Figure 2). Data represent the average of two independent experiments (Mean ± standard deviation [s.d.], *n* = 2). The initial titers ranged from 5 to 7 log_2_ and from 3 to 8 log_10_, respectively, for Ha activity and infectivity in BHK-21 cells.

Of the 15 isolates studied, five retained the initial Ha titer after 60 min at 56 °C (*K* = 0) (HL8, HL9, HL10, HL11, HL12), three lost one log_2_ within 40 min to 60 min (*K* = 2.23 to 4.05) (HL2, HL5 and HL13), another three lost two log_2_ between > 30 min to 60 min (*K* = 11.55 to 31.20) (HL3, HL4 and NB1) and four lost two or more log_2_ in ≤ 30 min (*K* = 35.84 to 59.74) (HL1, HL6, HL7 and NB2). The Ulster 2C reference strain lost 2 log_2_ in 45 min (*K* = 7.4).

Determination of 50% endpoint infectivity before and after heat treatment was carried out on BHK-21 cells. Seven isolates lost 2 or more log_10_ in ≤ 10 min at 56 °C (HL1, HL4, HL5, HL6, HL7, NB1 and NB2) (*K* = 27.37 to 175.97), four isolates between 10 min and 15 min (*K* = 43.75 to 90.28) (HL8, HL9, HL10 and HL13) and four isolates between 15 min and 30 min (*K* = 17.97 to 41.77) (HL2, HL3, HL11 and HL12). The Ulster 2C reference strain lost 2 log_10_ between 10 min and 15 min (*K* = 44.07).

Altogether, for eight of the 13 isolates from Huíla province, the time required for infectivity and Ha titer to decrease by two logarithmic orders at 56 °C was, respectively, > 10 min and > 30 min, therefore in accordance with the thermostability criteria proposed by Lomniczi (1975), these isolates have a phenotype I^+^Ha^+^ (HL2, HL3, HL8, HL9, HL10, HL11, HL12 and HL13). Two other isolates (HL4 and HL5) lost its infectivity titer by > 2 log_10_ in ≤ 10 min but sustained their Ha titer (< 2 log_2_ in > 30 min) and are classified as I^−^Ha^+^, while three other isolates (HL1, HL6 and HL7) rapidly lost both infectivity (> 2 log_10_ in ≤ 10 min) and Ha titer (> 2 log_2_ in < 30 min) therefore belonging to I^−^Ha^−^ phenotype. The two NDVs from Namibe province showed different phenotypes. Both isolates had a decrease in infectivity of 2 log_10_ in ≤ 10 min, belonging to phenotype I^−^, but while for NB1 isolate the Ha activity was maintained after 30 min (Ha^+^), NB2 isolate lost 2 log_2_ in Ha activity in < 30 min (Ha^−^). As expected, the lentogenic Ulster 2C reference strain showed a phenotype I^+^Ha^+^.

It has been known for a long time that, under the effect of heat, infectivity is usually more thermolabile than Ha activity (Pierce & Haywood 1973). In an attempt to explain the molecular basis of these phenotypes, several thermostable and thermolabile NDV strains were sequenced and compared. Mutation within the HN gene has been reported to contribute to the thermostability, phenotype and immunogenicity of ND virus (Kattenbelt, Meers & Gould 2006; Tan et al. 1995; Wen et al. 2013; Yusoff et al. 1996).

A more recent study, involving chimeric viruses generated by exchanging viral genes between a thermostable NDV strain and thermolabile LaSota strain using reverse genetics technology, revealed that the HN protein of NDV is a major determinant of thermostability (Wen et al. 2016). Based on this latter study, a fragment of the HN encoding gene from each isolate was sequenced, and nucleotide sequences obtained are available in GenBank (accession numbers MN918460 to MN918474). However, slight differences were observed among the sequences obtained, with most differences occurring between isolates HL4 and HL6 and the other isolates, which is not in accordance with what was observed in the thermostability studies, where HL4 and HL6 were considered as I^−^Ha^+^ and I^−^Ha^−^, respectively. Nevertheless, we may presume that all but four of the NDV isolates (HL1, HL6, HL7 and NB2) of this study are thermostable viruses.

The control and eradication of Newcastle disease only can be achieved following the implementation of surveillance and control programmes for the Newcastle disease virus. For this reason, similar to what has already been done in several developing countries (Jagne et al. 1991; Tu et al. 1998; Wambura, Kapaga & Hyera 2000), it is necessary to implement in Angola, surveillance and control programmes that are sustainable and easy to apply in the field. Their application can reduce the impact of ND on village and backyard chickens that are an important source of protein and income for families in rural and peri-urban areas in the country. It is therefore of utmost importance to promote vaccination campaigns against ND, preferably with the use of thermostable vaccines (Fanelli et al. 2022).

## Conclusion

Twenty-one out of 30 samples received from Huíla and two out of 31 samples from Namibe province tested positive to NDV by Lgene/RT-qPCR. All the samples from Huíla were from 2016, while the two samples from Namibe, one was collected in 2016 and the other in 2018. Newcastle disease virus was successfully isolated from 13 samples originating from Huíla province and two NDV samples from Namibe province.

Another study performed with the same samples revealed that the 15 isolates are from subgenotype VII.2 and have the cleavage site motif characteristic of virulent strains (^112^RRQKRF^117^). Moreover, the 2016 NDV isolate from Namibe province is related to an outbreak that affected the neighbouring country Namibia in that year, while the 2018 isolate is linked to the outbreak of the same year in the province of Huíla.

Following NDV thermostability and infectivity assays, and as expected, the lentogenic Ulster 2C reference strain showed a phenotype I^+^Ha^+^. Eight of the 13 isolates from Huíla province (HL2, HL3, HL8, HL9, HL10, HL11, HL12 and HL13) also have a phenotype I^+^Ha^+^, revealing to be good candidates for vaccine production.

The occurrence of ND in vaccinated birds raised the question of the antigenic distance between the NDV strains commonly used in commercial vaccines and the NDVs circulating in the geographic area where these vaccines were applied. The isolation, biological and molecular characterisation of NDV strains that currently circulate in Angola are a valuable source of thermostable NDVs. After evaluating the protection potential of the vaccine in use in vitro and/or in vivo, the eight thermostable strains of NDV of genotype I^+^Ha^+^ isolated in Angola and presented in this study can be genetically manipulated by reverse genetic technology in order to reduce their virulence and be adapted to develop more suitable vaccines to adopt in the country.
